# Malfunction of a Heimlich flutter valve causing tension pneumothorax: case report of a rare complication

**DOI:** 10.1186/1754-9493-4-8

**Published:** 2010-06-17

**Authors:** April O Paul, Chlodwig Kirchhoff, Michael V Kay, Albert Hiebl, Markus Koerner, Volker A Braunstein, Wolf Mutschler, Karl-Georg Kanz

**Affiliations:** 1 Munich University Hospital LMU, Department of Trauma Surgery - Campus Innenstadt, Nussbaumstrasse 20, D-80336 Munich, Germany; 2 Munich University Hospital LMU, Department of Radiology - Campus Innenstadt, Nussbaumstrasse 20, D-80336 Munich, Germany; 3 Plansafe GmbH, Landsberger Strasse 155, D-80336 Munich, Germany

## Abstract

**Background:**

Thoracic injuries play an important role in major trauma patients due to their high incidence and critical relevance. A serious consequence of thoracic trauma is pneumothorax, a condition that quickly can become life-threatening and requires immediate treatment.

Decompression is the state of the art for treating tension pneumothorax. There are many different methods of decompression using different techniques, devices, valves and drainage systems. Referring to our case report we would like to discuss the utilization of these devices.

**Case presentation:**

We report of a patient suffering from tension pneumothorax despite insertion of a chest drain at the accident scene. The decompression was by tube thoracostomy which was connected to a Heimlich flutter valve. During air transportation the patient suffered from cardiorespiratory arrest with asystole and was admitted to the trauma room undergoing manual chest compressions. The initial chest film showed a persisting tension pneumothorax, despite the chest tube that had been correctly placed and connected properly to the Heimlich valve. We assume that the Heimlich valve leaves did not open up and thus tension pneumothorax was not released.

**Conclusion:**

We would like to raise awareness to the fact that if a Heimlich flutter valve is applied in the pre-hospital setting it should be used with caution. Failure in this type of valve may lead to recurrent tension pneumothorax.

## Background

Thoracic injury plays an important role in major trauma patients as it occurs in 35 - 70% cases [1]. A serious consequence of thoracic trauma is pneumothorax, a condition that quickly can become life-threatening and requires immediate treatment. The incidence of pneumothorax due to thoracic injuries is estimated at 20% whereas the incidence of tension pneumothorax remains unclear [2]. Decompression is considered as the gold standard for treating tension pneumothorax. However, there are many different methods of decompression using different techniques, devices, valves and drainage systems. We herewith report a case of a defective Heimlich flatter valve used during resuscitation of a patient suffering from a traumatic cardiorespiratory arrest (TCRA) with persisting tension pneumothorax.

## Case presentation

At 10:45 the 68 year old, male patient got injured after being attacked by a bull while working at a rural slaughterhouse. 10 minutes after the accident, an advanced life support team arrived on scene. Initially, the patient was in severe respiratory distress due to flail chest. He was able to communicate and to respond to given commands but had no measurable blood pressure. Initial ECG monitoring showed ST elevations in the aVR lead. Emergency endotracheal intubation was performed and as the breathsounds over the left side were impaired, a tube thoracostomy was performed via a Monaldi approach in the 3^rd ^intercostal space midclavicular line and connected to a Heimlich flatter valve. Fluid resuscitation with cristalloids and colloids was initiated. Epinephrine and dopamine were then given as the response to fluid resuscitation was not sufficient. The patient was transferred in a critical condition 150 miles by helicopter from the rural district to the next level I trauma centre. At 12:10, during transport, the patient suffered from cardiorespiratory arrest with asystole. At 12:25 the patient was admitted to our trauma room undergoing manual external chest compressions with a massive subcutaneous emphysema despite the pre-hospital inserted chest tube, which had been inserted on the left side. Inspection of the tube and valve showed no obstruction through bending or clotted blood. Assuming a contralateral tension pneumothorax, a chest tube was placed on the right side while still on the gurney. Prior auscultation of the breath sounds was not possible due to the massive emphysema. An immediate chest film was taken the moment, when the patient was placed on the radiotranslucent trauma room table. Focused assessment with ultrasound in trauma (FAST) revealed neither pericardial effusion nor massive free abdominal fluid. A left side resuscitative thoracotomy was performed for direct cardiac massage and thoracic aortic occlusion. After opening the thorax it could be seen that the chest drain with the connected Heimlich flutter valve had been placed correctly in the pleural space.

**Figure 1:**
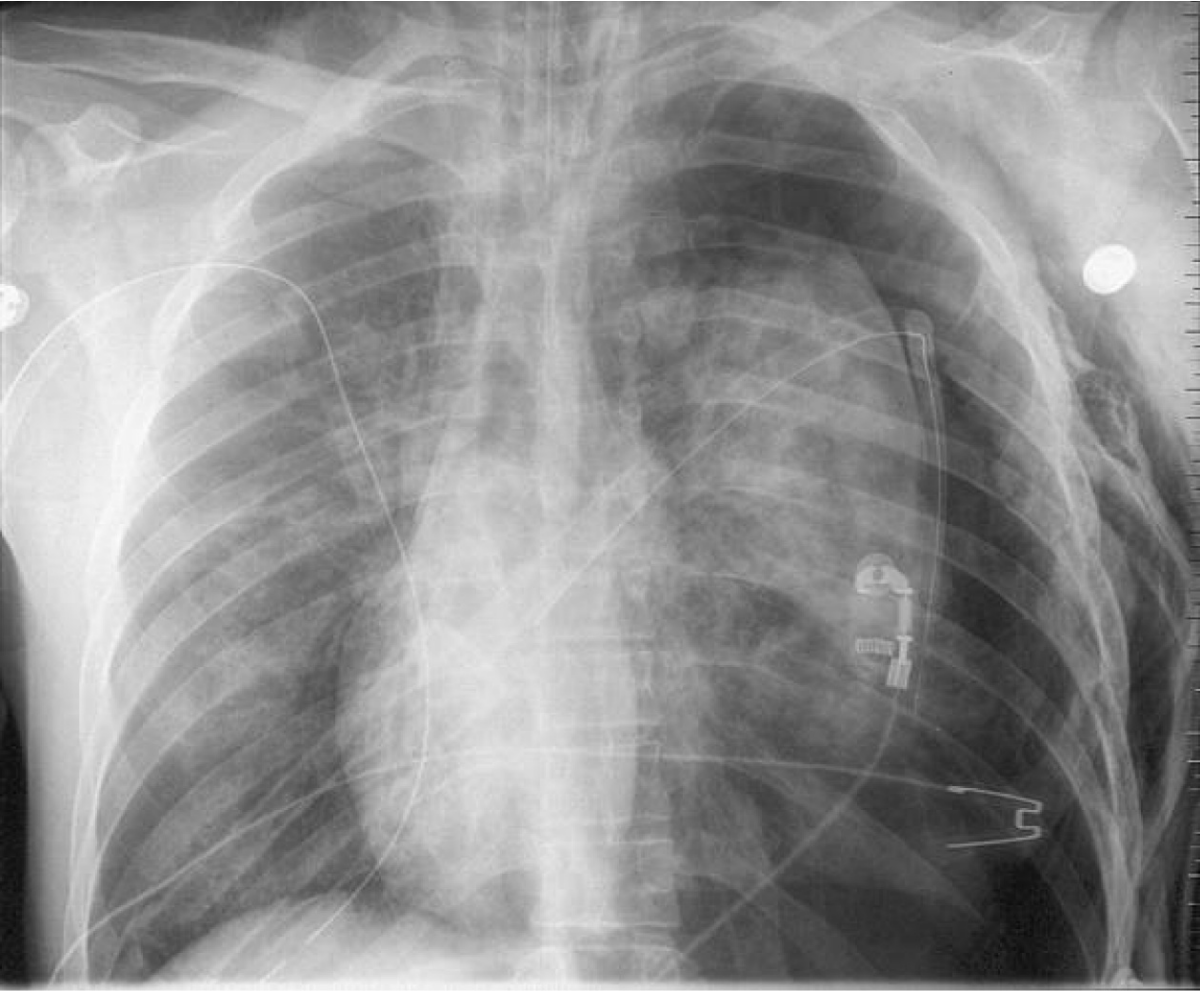
**Chest film revealing persisting tension pneumothorax on the left side despite the correctly placed chest tubes on both sides**. Furthermore showing a massive subcutaneous emphysema also on the left side.

Now available developed chest film demonstrated correct tube positioning corresponding to the in situ findings. A subcutaneous emphysema but also a massive tension pneumothorax on the left side was visible, despite the inserted chest tube on scene [figure 1]. Further exploration of the thorax showed an insufficient filling of the ventricles, an apical lung rupture and comminuted multiple rib fractures. Under manual cardiac massage, catecholamine adminstration and volume restoration, ventricular flutter occurred and was successfully defibrillated to a sinus rhythm with a blood pressure of 100 mmHg. Initial blood samples revealed hemoglobine concentration of 7,2 mg/dl, thromboplastine time of 31% and base excess of -22,5.

The apical lung rupture on the left side was sutured and 2000 ml of blood, resulting from multiple rip fractures, was drained via a chest tube on the right side. In the further progress celiotomy was performed and abdominal exploration showed no additional relevant findings.

Despite the occluded thoracic aorta, high-dosage catecholamine medication and massive blood transfusion with 20 units of packed red blood cells, the patient remained hemodynamically unstable. Although supportive manual cardiac massage was performed, cardiac output detoriated and intermittent ventricular flutter episodes occurred. Resuscitative efforts were stopped at 13:45. Post mortem computed tomography and autopsy confirmed our major findings and revealed, except a pelvis fracture, no further relevant injuries.

## Discussion

Tension pneumothorax is a life-threatening injury which needs to be detected quickly and treated appropriately. In a recently published study of our group, we found out that chest decompression on scene seem to have a strong positive impact on probability of survival in traumatic cardiorespiratory arrest (TCRA) [3]. It is therefore important to have reliable parameters with respect to detection of tension pneumothorax in the pre-hospital setting. Waydhas et al. reviewed current literature regarding the diagnosis of tension pneumothorax. According to widespread expert opinion, loss of breath on the injured side, signs of life-threatening haemodynamic and respiratory compromise are seen as reliable clinical signs to diagnose a tension pneumothorax [4].

In the pre-hospital setting decompression of the pleural space is performed either by needle decompression, surgical decompression or by tube thoracostomy.

The advantage of decompression by the use of a needle is speed, use of readily available material and the simple procedure [5]. Because of the narrow lumen, resulting in insufficient decompression, additional chest tube insertion may be required in a significant number of patients [6].

A surgical decompression is more effective but in terms of release of air it needs to be considered that the incision tends to close again spontaneously leading to recurrent tension pneumothorax and therefore requires close monitoring[4].

Chest tube insertion is considered an effective method to decompress tension pneumothorax [7]. However it can result in complications such as injury of the lung, heart and abdominal organs [8-10]. Failure rate due to malposition is reported to be 11.2% [11]. For the lateral approach, injury to an intercostalartery, stenosis of the subclavian artery, injury to the vena cava inferior, perforation of a lung, perforation of the right atrium, of the right or left ventricle, Horner's syndrome and intraabdominal malposition have been reported [8-10,12-17]. Considering the ventral approach injury to the heart, to the oesophagus, mediastinum, induction of a contralateral pneumothorax, injury to the phrenic nerve, and an arteriovenous fistula have been described [9,11,18,19]. In a prospective study our group showed that both approaches, the ventral Monaldi (2.-3. intercostal space) and the lateral Buehlau (4.-6. intercostal space), could be performed [20]. Considering malfunction of a chest tube due to malposition no statistical significant difference between the two different approaches could be identified.

Our presented case leads to the question which drainage system should be applied in terms of pre-hospital management of tension pneumothorax. Common devices are closed bag or collection chamber systems with or without an incorporated air or water seal or the often used Heimlich flutter valve.

With regard to choosing a drainage system containing of a valve or not it is important whether the patient is breathing spontaneously or positive pressure ventilated by respirator. If the patient is breathing spontaneously a vent is necessary. Whereas if the patient is positive pressure ventilated the pressure within the lung prevents it from collapsing and no vent is necessary.

A closed bag system can only be used under conditions that are well controlled. The bag does not have a valve and therefore bears the risk of recurrent tension pneumothorax due to increasing pressure in the collecting system caused by a persisting air leak. As explained above, this system would be appropriate for the use in positive pressure ventilated patients.

The water seal is an efficient drainage system but has no major significance regarding its use in the field, as it is difficult to apply in this environment, especially with respect to transport. Water seals only work in an upright position, during resuscitation and transport they tend to tilt. Consequently they are used mainly in the hospital in both, spontaneous breathing and ventilated patients.

The Portex chest drainage system consists of a bag incorporating a one way flutter valve and a vented outlet. This kind of drainage system allows for collection of pleural secretion without further risk of recurrent tension pneumothorax as it is connected to a valve; provided the system is used correctly. The producer recommends to open up the valve before use, by injecting 20 ml of air, to assure that the valve is working safely. However, the Portex system, like the water seal, only works properly in an upright position. Graham et al. showed in a prospective randomized study with patients undergoing thoracotomy that these drainage bags were as effective in draining the chest of blood as an underwater seal, and as effective at releasing air from the chest unless pleural suction was required. There were no cases of blockage with blood in contrast to reported incidents with the Heimlich valve [21].

The Heimlich flutter valve has been developed for the release of pneumothorax and can be used in patients who are breathing spontaneously. It is constructed of a rubber tubing and is encased by a transparent plastic chamber which can be connected on one side to the chest drain and on the other side to a drainage system. The rubber tubing is compressed on one side to form leaflets that control unidirectional flow. The advantage of the Heimlich valve is that as it is easy to apply as well as to transport. Using a Heimlich valve in the hospital setting enables a shorter drainage time and hospitalisation [22].

The Asherman Chest Seal (ACS) is a sterile occlusive dressing with a one-way Heimlich valve for treating open pneumothorax in the acute settings. The ACS is a standard device for the US Army, the British Army and the US Navy for emergencies in the battlefronts for air leaks in the chest. Paramedics use it frequently in acute management of pneumothorax [23]. Furthermore the ACS can be applied in the clinical setting as shown by Rathinam et al. [24]. They used the seal in patients with persistent air leak after thoracic surgery and emphasized that it is imperative to check that the valve is working before application. Although the ACS and the Heimlich valve are similar, the ACS does not have an intra- thoracic component. Literature reports failure of the Heimlich flatter valve due to coagulated blood blocking the device and spontaneous dysfunction of the valve leading to recurrent tension pneumothorax similar to our presented case [4,25,26]. Furthermore Spouge et al published a case report describing a tension pneumothorax after reversal of a Heimlich valve [27].

Hiebl did an experiment in his doctoral thesis in which he tested 16 Heimlich flutter valves stored in ambulances and helicopter of the Munich EMS [28]. These valves were randomly sampled and after being removed from the ambulances or helicopter replaced by new valves. Eight of the devices were already expired and eight were still valid. Each valve has been connected to an upright tube of 200 cm height which was filled with water. Standard opening pressure for the valves was defined as 5 cmH2O [29]. Two of the 8 (25%) valid valves did not open up at 5 cm H2O and 7 of the 8 (87.5%) valves with an exceeded expiration date (p = 0.04 Fisher exact test). 3 of the expired valves were still occluded when applying an opening pressure of 200 cm H2O so that they never opened up at all. Although a number of 16 Heimlich valves is not representative it still indicates that the leaves of the valve tend to adhere, especially after they reached the expiration date recommended by the manufacturer. This needs to be taken into consideration since Heimlich valves are rarely used in the daily routine and therefore tend to be stored for a long time on ambulance or helicopter. Consequently the expiration date needs to be checked before usage as there is a significant correlation between malfunction and expiration date.

**Figure 2:**
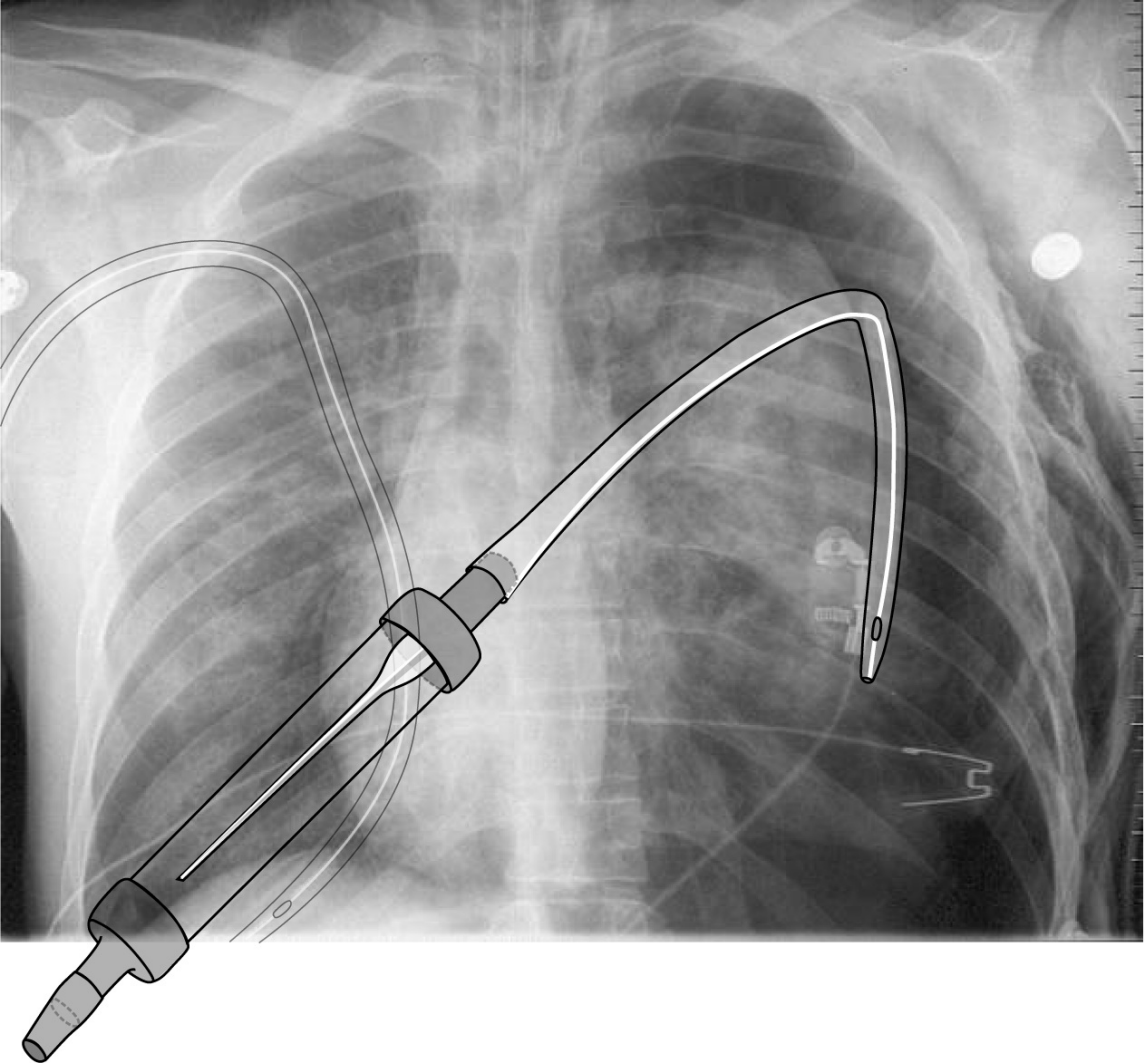
**Chest film with the graphical representation of the chest tube within pleural cavity and the correctly connected flutter valve**.

The chest film on admission demonstrated that there was a persisting tension pneumothorax on the left side although the chest drain was inserted correctly and connected the right way to the Heimlich valve which was proven by the radiography [figure 2]. This was also shown by the findings during thoracotomy.

Consequently it must be due to a malfunction of the Heimlich flatter valve that there was no release of the tension pneumothorax. As there was initially no blood in the chest tube on the left side, an occlusion due to coagulated blood cannot be considered. In our case we suspect that the rubber tubing leaflets did not open up. We assume that the leaflets got sticked together during storage in the helicopter. It is difficult to say which impact the malfunction of the valve has with regard to the outcome in our patient but one could certainly consider it as an important factor triggering the traumatic cardiorespiratory arrest. Other factors which must be taken into consideration are the severe thoracic trauma itself, the prolonged pre-hospital time interval and the critical condition on admission.

## Conclusion

Coming to a conclusion we would like to raise awareness to the fact that if a Heimlich flutter valve is applied in the pre-hospital setting it should be used with caution. Failure in this type of valve may lead to recurrent tension pneumothorax. In patients who are a breathing spontaneously it is however necessary to apply a drainage system with a valve to prevent an open pneumothorax. In this case we would recommend to choose other systems. In patients who are ventilated with positive pressure we would prefer to use of a simple closed bag system without any kind of valve. In this case it is essential to carefully monitor the position, filling and pressure of the bag, and to release any overpressure just by intermittently disconnecting the bag from the tube.

## Competing interests

The authors declare that they have no competing interests.

## Authors' contributions

AOP, KGK and CK participated in the idea, planning and writing the report. MVK, AH, MK, VAB and WM participated in collecting information on the topic and writing the report.

All authors have seen and approved the final version of the manuscript.

## Consent

Written informed consent was obtained for publication of this case report and any accompanying images. A copy of the written consent is available for review by the Editor-in-Chief of this journal.
